# Mental well-being of medical students and the need for mental health care in the dynamics of 2020-2024 years

**DOI:** 10.1192/j.eurpsy.2025.1579

**Published:** 2025-08-26

**Authors:** E. Chumakov, I. Pchelin, P. Makarova, N. Petrova

**Affiliations:** 1 Department of Psychiatry and Addiction; 2 Department of Faculty Therapy, St. Petersburg State University, St. Petersburg, Russian Federation

## Abstract

**Introduction:**

Research has consistently highlighted the vulnerability of medical students to poor mental health and wellbeing. The COVID-19 pandemic has further exacerbated this issue. However, there is limited understanding of medical students’ mental wellbeing dynamics after the pandemic.

**Objectives:**

The aim of the study was to comparatively analyse the mental wellbeing and mental health needs of medical students in comparison with those during the COVID-19 pandemic.

**Methods:**

An anonymous structured online survey was conducted among students of a medical institute in St. Petersburg, Russia. The sample included responses from 152 students (76.3% women) of all courses of study. The results were compared with the data of a survey conducted at the same institute in 2020 (Chumakov *et al*. Middle East Curr Psychiatry 2021;28, 38).

**Results:**

The majority of respondents (n=145; 95.4%) reported experiencing significant stress in their lives (95.8% in 2020). The main sources of stress included education-related factors (83.6%), uncertainty about the future (72.4%), financial problems (48.0%), intimate/family relationships (46.7%), work (27.6%), and housing problems (19.1%). Thirteen students (8.6%) reported that they had been diagnosed with a mental health disorder prior to enrolling in the institute (6.1% in 2020; p=0.393). Twice as much students (n=27; 17.8%) were diagnosed with mental disorders during institute studies (15.2% in 2020; p=0.53). The mental disorders reported by the respondents were dominated by depressive disorders (n=7), anxiety disorders (5), mixed anxiety and depressive disorder (5), ADHD (5), bipolar disorder and cyclothymia (3). At the time of the study, 26 students (17.1%) were being seen by a mental health professional (10.9% in 2020; p=0.111), the same number of students were taking any prescribed medication for mental health (10.9% in 2020; p=0.111). One-third of respondents (n=53; 34.9%) had taken non-prescription medication in the last year to improve their well-being or mood (27.3% in 2020; p=0.143), and 45 (29.6%) had taken medication in the last year to improve concentration or academic performance (38.3% in 2020; p=0.107).

**Conclusions:**

The study showed high mental health care needs among medical students with a tendency to self-medication. Notably, mental health indicators have not improved since 2020 despite the end of the COVID-19 pandemic. Our findings highlight consistent trends in medical students’ mental health and underscore the need for targeted interventions to support this vulnerable population.

**Disclosure of Interest:**

None Declared

